# Dynamics of the Pacific oyster pathobiota during mortality episodes in Europe assessed by 16S rRNA gene profiling and a new target enrichment next‐generation sequencing strategy

**DOI:** 10.1111/1462-2920.14750

**Published:** 2019-07-31

**Authors:** Aide Lasa, Andrea di Cesare, Giovanni Tassistro, Alessio Borello, Stefano Gualdi, Dolors Furones, Noelia Carrasco, Deborah Cheslett, Amanda Brechon, Christine Paillard, Adeline Bidault, Fabrice Pernet, Laura Canesi, Paolo Edomi, Alberto Pallavicini, Carla Pruzzo, Luigi Vezzulli

**Affiliations:** ^1^ Department of Earth, Environmental and Life Sciences (DISTAV) University of Genoa Genoa Italy; ^2^ Department of Microbiology and Parasitology Universidade de Santiago de Compostela Santiago de Compostela Spain; ^3^ National Research Council‐Water Research Institute (CNR‐IRSA), Largo Tonolli 50, 28822 Verbania Italy; ^4^ Department of Plant and Microbial Biology University of Zürich Zürich Switzerland; ^5^ IRTA, Sant Carles e la Ràpita Tarragona Spain; ^6^ Fish Health Unit The Marine Institute, Rinville Oranmore Galway Ireland; ^7^ Laboratoire des sciences de l'Environnement Marin, Institut Universitaire Européen de la M Université de Bretagne Occidentale – UMR6539 CNRS/UBO/IRD/Ifremer Plouzané France; ^8^ Ifremer, Physiologie Fonctionnelle des Organismes Marins UMR 6539 LEMAR (CNRS/Ifremer/IRD/UBO) Technopole Iroise, CS 10070 29280 Plouzane France; ^9^ Department of Life Sciences University of Trieste Trieste Italy

## Abstract

Infectious agents such as the bacteria *Vibrio aestuarianus* or *Ostreid herpesvirus 1* have been repeatedly associated with dramatic disease outbreaks of *Crassostrea gigas* beds in Europe. Beside roles played by these pathogens, microbial infections in *C*. *gigas* may derive from the contribution of a larger number of microorganisms than previously thought, according to an emerging view supporting the polymicrobial nature of bivalve diseases. In this study, the microbial communities associated with a large number of *C*. *gigas* samples collected during recurrent mortality episodes at different European sites were investigated by real‐time PCR and 16SrRNA gene‐based microbial profiling. A new target enrichment next‐generation sequencing protocol for selective capturing of 884 phylogenetic and virulence markers of the potential microbial pathogenic community in oyster tissue was developed allowing high taxonomic resolution analysis of the bivalve pathobiota. Comparative analysis of contrasting *C*. *gigas* samples conducted using these methods revealed that oyster experiencing mortality outbreaks displayed signs of microbiota disruption associated with the presence of previously undetected potential pathogenic microbial species mostly belonging to genus *Vibrio* and *Arcobacter*. The role of these species and their consortia should be targeted by future studies aiming to shed light on mechanisms underlying polymicrobial infections in *C*. *gigas*.

## Introduction

Recent advances in DNA sequencing technology is enabling new quantitative insights into the microbial community diversity associated with human and animal tissues (Yatsunenko *et al*., 2012; Yarza *et al*., 2014). It is now recognized that host‐associated microbial communities (also named the microbiota) (Lederberg and McCray, 2001) are playing an important role in animal health by providing prominent services ranging from nutrient processing to protection from diseases. Marine bivalves host high microbial abundance and diversity and alteration of the microbiota due to stressful conditions and/or environmental changes was previously linked with a condition of a compromised health status and susceptibility to diseases (Lokmer and Wegner, 2015).

Diseases affecting the Pacific oyster (*Crassostrea gigas*) have been rising over the past decades, representing a significant threat for commercial exploitation of both farmed and natural stocks (Alfaro *et al*., 2018). In Europe, mass mortality episodes of *C*. *gigas* in farming areas are attributed to complex interactions among oysters, microbial pathogens and environmental variables (Pernet *et al*., 2012). In particular, stressful environmental conditions, such as warmer seawater temperatures, were observed to favour a shift in *C*. *gigas* bacterial communities toward pathogen‐dominated communities also promoting colonization by secondary opportunistic pathogens and non‐resident microbial species (Lemire *et al*., 2015; Lokmer and Wegner, 2015).

Mortality of *C*. *gigas* spat and juvenile is mainly associated with infection by the herpes‐like virus Oyster Herpesvirus type 1 (OsHV‐1) usually when water temperature reaches 16°C (Segarra *et al*., 2010). Infected oysters show a reduction in feeding and swimming activities and mortality can reach 100% in a few days. In contrast, mortality of adult oysters in Europe has been mainly reported in association with the detection of the bacterium *Vibrio aestuarianus* (Travers *et al*., 2015). In this case, infection seems to last for a long period, reaching a cumulative mortality rate up to ~30% at the end of the farming cycle.

Although these pathogens have been identified to play a role in oyster diseases, there is an emerging view that microbial infections, in marine bivalves, may derive from the contribution of different microbial species/strains that act as a ‘community of pathogens’ (hereafter referred to as the ‘pathobiota’) rather than a single species/strain as the only etiological agent (Lemire *et al*., 2015; de Lorgeril *et al*., 2018). Under this perspective evidence has been provided supporting the view that oyster infections might be seen as infectious disorders caused by the contribution of a larger number of pathogens (e.g. populations or consortia) than previously thought (Lemire *et al*., 2015). The question now is no longer whether microorganisms are involved in the pathogenesis of such diseases, but which specific microbial species or strains are involved.

Although addressing this issue will be of great value in improving the general understanding of bivalve diseases and to drive future studies to unravel their mechanisms, answering the above question is not straightforward.

In fact, accurate phylogenetic analysis of the bivalve microbiota, including the potential pathogenic community, has been historically hampered by methodological constrains, such as the use of traditional culture‐dependent protocols, as a large fraction of the bivalve‐associated microbial community (e.g. unculturable bacteria) may go undetected using these methods (Garnier *et al*., 2007). More recently, 16S rRNA gene‐based analysis of the microbial diversity, based on next‐generation sequencing protocols, was employed for taxonomic identification of bivalve‐associated bacteria (Lokmer and Wegner, 2015; Lokmer *et al*., 2016). However, although this approach allows us to overcome the cultivation bias, 16S rRNA profiling could not be successfully applied for high taxonomic resolution analysis of the microbial community (e.g. taxonomic resolution to the level of species or strains) due to the lack of phylogenetic value of the 16S rRNA gene for many bacterial groups (Rajendhran and Gunasekaran, 2011). This is particularly true for bacteria belonging to the *Vibrio* genus that are thought to play a primary role in oyster infections and mortality outbreak (Vezzulli *et al*., 2015). The same holds true for shotgun metagenomic techniques due to difficulties in separation of host DNA from microbial DNA resulting in non‐optimal detection and taxonomic resolution of microbial species. Thus, investigating polymicrobial nature of OsHV‐1 disease and the unknown nature of V. aestuarianus infection affecting *C*. *gigas* deserve new tools.

The aim of this study was to overcome these constrains and be able to provide new insights into the high‐level (species) taxonomic composition of the oyster pathobiota in *C*. *gigas* samples collected during mortality outbreaks in Europe. To this end, a new target enrichment next‐generation sequencing approach was developed and applied to target 884 phylogenetic and virulence markers of the potential pathogen community associated to bivalve tissues. 16S rRNA gene profiling of the microbial community was also conducted on a large number of contrasting (e.g. infected vs not infected) *C*. *gigas* samples to support the ‘pathobiota’ analysis and to extensively investigate the patterns and dynamics of oyster microbiota during mortality events.

## Results

### *OsHV‐1 and* V. aestuarianus *detection in* C. gigas *samples*

A total of 525 *C*. *gigas* samples collected from 2016 to 2017 in three European sites, i.e., Ebro delta, Dungarvan Bay and Bay of Brest (Supplementary Material and Methods, Fig. S1, Table S1) were screened for the presence of OsHV‐1 and *V*. *aestuarianus* using quantitative real‐Time PCR (Table 1). In general, samples collected during oyster mortality episodes tested positive for at least one of the two pathogens suggesting a strong link between presence of these microorganisms in the oyster tissues and development of disease (Labreuche *et al*., 2010; Segarra *et al*., 2010). In particular, *V*. *aestuarianus* was found associated to adult *C*. *gigas* mortality observed in the Ebro delta on 13th April 2016 and 31st May 2017 and in Dungarvan Bay on 3rd October 2016 (Table 1). OsHV‐1 DNA was detected in spat *C*. *gigas* sampled during 12 mortality episodes in the Ebro delta, Dungarvan Bay and Bay of Brest (Table 1).

**Table 1:** *Crassostrea gigas* samples collected at different European sites and analysed in this study (*n* + number of samples/total number of analysed samples scoring positive to species‐specific qPCR targeting the pathogen *V*. *aestuarianus* or OshV‐1 in oyster tissues).

Sampling date	Code	Age	Origin	Ploidy	Mortality (%)	OshV‐1 (Real‐time PCR)	*V. aestuarianus* (Real‐time PCR)
*Ebro Delta (Spain)*							
13_04_2016	EbDva13apr16/EbH13apr16	Adult	Nature	Diploid	23% (Up to 50%)	0+/30	22+/30
26_04_2016	EbDoshV26apr16(c3)/EbH26apr16(c4)	Spat	Nature	Diploid	76% (Up to 90%)	17+/30	0+/30
26_04_2016	EbDoshV26apr16(c5)/EbDva26apr16(c6)/EbH26apr16(c6)	Spat	Nature	Diploid	46.6% (Up to 80%)	21+/30	4+/30
19_07_2016	EbDoshV19jul16/EbH19jul16	Spat	Hatchery	Diploid	3% (light mortality)	1+/30	0+/30
19_07_2016	EbH19jul16(c2)	Spat	Nature	Diploid	No mortality	0+/30	0+/30
24_11_2016	EbH24nov16/EbDoshV24nov16/EbDva24nov16	Spat	Nature	Diploid	30% recent mortality	7+/30	1+/30
25_01_2017	EbH25jan17	Spat	Hatchery	Diploid	No mortality	0+/30	0+/30
26_01_2017	EbH26jan17	Spat	Hatchery	Diploid	No mortality	0+/30	0+/30
05_05_2017	EbDoshV5may17/EbH5may17	Spat	Hatchery	Triploid	87.83% mortality	10+/18	0+/18
05_05_2017	EbH5may17(c1)	Spat	Hatchery	Triploid	No mortality	0+/30	0+/30
31_05_2017	EbDva31may17/EbH31may17	Spat	Hatchery	Triploid	85% mortality	0+/30	9+/30
*Dungarvan Bay (Ireland)*							
05_07_2016	DrDva7jul16/DrH7jul16	Adult	Hatchery	Triploid	20%	0+/30	2+/30
05_07_2016	DrDoshV7jul16SPAT/DrDva7jul16SPAT/DrH7jul16SPAT	Spat	Hatchery	Triploid	70%–100%	25+/30	3+/30
03_10_2016	DrDva3oct16/DrH3oct16	Adult	Hatchery	Triploid	End of mortality	0+/30	11+/30
03_10_2016	DrDoshV3oct16SPAT/DrDvaV3oct16SPAT/DrH3oct16SPAT	Spat	Hatchery	Triploid	End of mortality	12+/30	1+/30
10_01_2017	DrH10jan17	Adult	Hatchery	Triploid	No mortality	0+/30	0+/30
10_01_2017	DrH10jan17SPAT	Spat	Hatchery	Triploid	No mortality	0+/30	0+/30
*Bay of Brest (France)*							
10_07_2017	BbDoshV10jul17/BbDva10jul17/BbH10jul17	ad/sp/jv	Hatchery	Diploid	Mortality	2+/12	2+/12
05_10_2017	BbDoshV5oct17/ BbDva5oct17/BbH5oct17	ad/sp/jv	Hatchery	Diploid	No mortality	3+/15	1+/15

Code legend = [Eb, Ebro delta; Dr, Dungarvan bay; Bb, Bay of Brest]/[D, Infected; H, Not Infected]/[va, *Vibrio aestuarianus*; oshV, *Ostreid herpesvirus* 1]/[collection date].

### *Microbiota analysis*

One hundred one contrasting *C*. *gigas* samples were selected in the Ebro Delta (*n* = 50), Dungarvan bay (*n* = 40) and the Bay of Brest (*n* = 11) for microbiota analysis based on results from PCR screening analysis. In the experimental design, most interesting sampling periods were chosen (e.g. periods with high mortality or absence of mortality), and for each period, 10 contrasting individual oyster samples (five samples infected by OsHV‐1 or *V*. *aestuarianus* and five non‐infected control samples) were selected. Sequencing of the samples by Ion Torrent technology produced more than 14.000.000 amplicon sequence reads spanning the V4 hypervariable region of the bacterial 16S rRNA gene. Barcode and adapters sequences were removed and raw sequences were trimmed to minimize bias associated with PCR amplification of target genes. In particular, reads that contained one or more ambiguous bases, had errors in the barcode or primer sequence, were atypically short (100 bp), and had an average quality score <0.05 were removed from the data set. This process produced 13.143.153 trimmed reads corresponding on average to 146.000 sequence reads per analysed sample.

**Figure 1:**
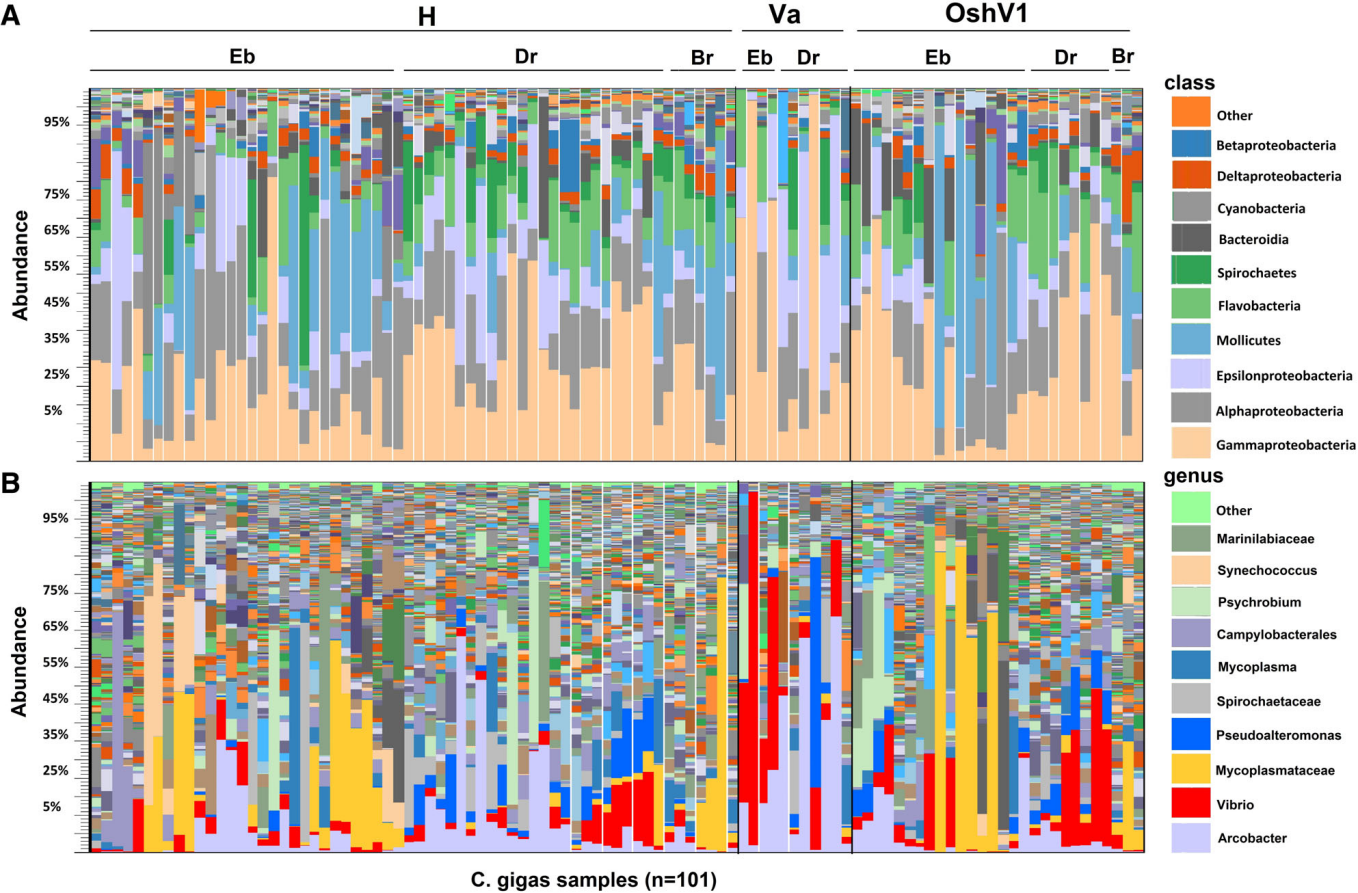
Taxonomical composition at the class (A) and genus (B) level of 101 *C*. *gigas* microbiota grouped by oyster health status (H, not infected oysters; Va, *Vibrio aestuarianus*‐infected oysters; OshV1, Ostreid herpesvirus 1‐infected oysters) and geographic locations (Eb, Ebro delta; Dr, Dungarvan bay; Br, Bay of Brest), as revealed by sequencing of the V4 hypervariable region of the 16S rRNA gene. [Color figure can be viewed at wileyonlinelibrary.com]

**Figure 2:**
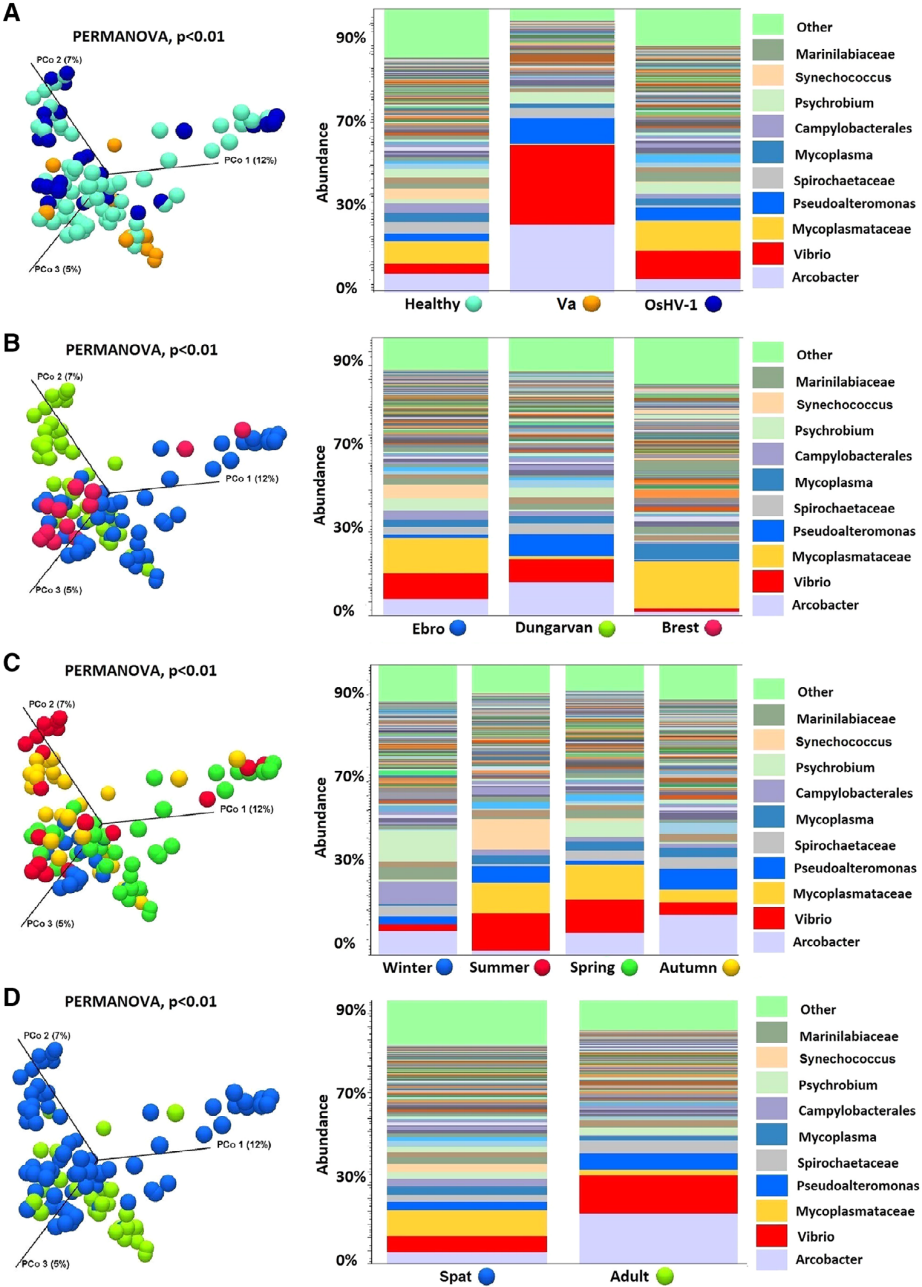
Comparative analysis of *C*. *gigas* microbiota (Beta diversity analysis) under different conditions: health status (A), geographic location (B), season (C) and animal age (D) (Va, *Vibrio aestuarianus*‐infected oysters; OshV1, Ostreid herpesvirus 1‐infected oysters). [Color figure can be viewed at wileyonlinelibrary.com]

In general, the composition of the bacterial community associated to *C*. *gigas* was dominated by the classes of *Gamma* and *Alphaproteobacteria* accounting on average for 28% and 15% of the total bacterial diversity followed by *Epsilonproteobacteria* (11%), *Mollicutes* (10%) and *Flavobacteria* (9%) (Fig. 1). Rarefaction curves computed for total operational taxonomical units (OTUs) abundance almost reached a plateau indicating that sequencing effort was good enough to describe the majority of phylotypes in most of the samples (Fig. S2). Generally, alpha diversity was lower in adult oysters infected by *V*. *aestuarianus* whilst it was higher in oyster spat infected by OsHV‐1 (Fig. S2). Different physiological or environmental conditions including animal health status and age, geographic location and season significantly influenced the composition of the oyster microbiota as showed by beta diversity analysis and PERMANOVA testing (*p* < 0.01) (Fig. 2). Analysis of contrasting bivalve samples based on the presence/absence of microbial pathogens showed that a significant shift in the microbiota community was observed in adult oyster infected by *V*. *aestuarianus* (Fig. 2). In particular, an increase in abundance of bacteria belonging to the genus *Vibrio* and *Arcobacter* was observed in *V*. *aestuarianus*‐infected oysters compared to either *V*. *aestuarianus* non‐infected or OsHV‐1 infected oysters. In oyster spats infected by OsHV‐1 an increase in the *Vibrio* fraction was also observed compared with non‐infected oysters.

The analysis of the core microbiota showed that the microbial community of oyster samples is dominated by two main bacterial groups, namely *Vibrio* and an uncultured bacterial group (uncultured‐053) (Supplementary Material and Methods, Table S2). When analysing adult *C*. *gigas* samples infected with *V*. *aestuarianus*, core taxa were assigned to *Arcobacter* and *Vibrio* genera. In contrast, core taxa of *V*. *aestuarianus* non‐infected samples were assigned to the uncultured‐053 group. When OsHV‐1 DNA was detected in spat oysters, the core microbiota included *Vibrio* but also the uncultured‐053 group. Interestingly, the core microbiota of oyster spats and adult was clearly different. While *Vibrio* and *Arcobacter* were members of the core microbiota in adult oysters, in oyster spats the core group was dominated by *Vibrio*, uncultured‐053, *Haliea* and *Marinicella*. Differences in the core microbiota were also evident across the different geographic locations. In oysters from Dungarvan bay the core groups were *Sulfitobacter*, uncultured‐053, *Arcobacter*, *Pseudoalteromonas*, *Marinicella*, *Vibrio* and *Borrelia*, while in Ebro Delta the core microbiota was formed only by *Vibrio* and uncultured‐053. On the other hand, the core microbiota of the samples from the Bay of Brest was more diverse than that of oysters from other sites, with more than 20 different bacterial groups (*Arcobacter*, *Vibrio*, *Mycoplasma*, *Winogradskyella*, *Haliea*, *Sulfitobacter*, *Polaribacter*, *Marinicella*, *Lutimonas*, *Aquibacter*, *Roseovarius*, uncultured‐104, uncultured‐079, uncultured‐053, uncultured‐015, OM60(NOR5) clade, uncultured bacterium‐088, uncultured‐072, uncultured‐050 and uncultured bacterium‐248) probably reflecting the smaller number of samples analysed in this site. Seasonality also affected the composition of the core microbiota, and a transition of specific genera was identified (e.g. *Sulfitobacter* and *Marinicella* from cold seasons to warmer seasons) (Supplementary Material and Methods, Table S2).

### *Pathobiota analysis*

A target enrichment next‐generation sequencing protocol was for the first time applied for high taxonomic resolution analysis of the bivalve pathobiota on a total of 12 selected contrasting *C*. *gigas* samples collected during mortality episodes (e.g. *C*. *gigas* samples infected and non‐infected by *V*. *aestuarianus* or OsHV‐1) and in the absence of mortality. A mock community sample (positive control) and a nuclease‐free water sample (negative control) were also included in the analysis (see methods section for details). To run the protocol, a ‘pathobiota’ sequence database containing 884 phylogenetic and virulence markers of the bivalve microbial pathogen community was built and used to produce a total of 12.114 biotinylated RNA baits for selective capturing of target DNA via hybridization as described in the methods section (Table 2). Sequencing of enriched DNA libraries produced a total of 67.614.544 sequence reads corresponding to about 5.634.545 reads for each sample. On average, less than 5% of the reads mapped against reference sequences from the pathobiota database and were used to produce consensus sequences for pathobiota taxonomic classification with the NCBI BLAST function.

**Table 2:** Phylogenetic and virulence markers targeted by the target enrichment NGS protocol to study the potential microbial pathogenic community (pathobiota) in *C*. *gigas* tissues (Rosenberg *et al*., 2007; Travers *et al*., 2015).

Target	Taxonomy	Main host	Marker gene	No. of allelic variants	Length (nt)
*Vibrio* spp.	*Vibrio* spp.	Human, Animal	*gyrB*	243	400
	*Vibrio* spp.	Human, Animal	*recA*	204	400
	*Vibrio* spp.	Human, Animal	*atpA*	133	400
	*Vibrio* spp.	Human, Animal	*dnaJ*	56	400
	*Vibrio* spp.	Human, Animal	*pyrH*	113	400
	*V*. *tasmaniensis*	*Crassostrea gigas*	LGP32 probes	10	400
	*V*. *cholerae* O1 El Tor	Human	*ctxA*	1	777
	*V*. *cholerae* O1 El Tor	Human	*ctxB*	1	375
	*V*. *cholerae* O139	Human	*ctxA‐B*	1	938
	*V*. *cholerae* O1 el Tor	Human	*tcpA*	1	675
	*V*. *cholerae* O1 classical	Human	*tcpA*	1	675
	*V*. *cholerae* O1 el Tor	Human	*rstR*	1	339
	*V*. *cholerae* O1 classical	Human	*rstR*	1	336
	*V*. *cholerae* O139	Human	*wbf*	1	449
	*V*. *cholerae* O1 el Tor	Human	*gbpA*	1	400
	*V*. *parahaemolyticus*	Human	*toxR*	1	552
	*V*. *parahaemolyticus*	Human	*tdh*, *trh*	3	570
	*V*. *vulnificus*	Human	*vvhA*	1	1416
	*V*. *vulnificus*	Human	*rtxA1*	1	400
	*V*. *tasmaniensis*	*Crassostrea gigas*	*vsm* (LGP32 strain)	1	1824
	*V*. *tasmaniensis*	*Crassostrea gigas*	*ompU* (LGP32 strain)	1	400
	*V*. *aestuarianus*	*Crassostrea gigas*	*vam*	1	1836
	*V*. *tapetis*	*Ruditapes philippinarum*	*djlA*	1	1826
	*V*. *coralliilyticus*	*Paramuricea clavata*	*vcpA*	15	1824
	*V*. *harveyi*	Stony corals	*vhhA*	1	1260
	*V*. *crassostreae*	*Crassostrea gigas*	*R‐5*.*7*	1	2397
	*V*. *tubiashii*	*Crassostrea gigas*	Metalloprotease	1	1821
	*Vibrio* spp.	Human, Animal	*MSHA*	12	400
*Arcobacter* spp.	*Arcobacter* spp.	Human	*gyrB*	27	400
*Nocardia crassostrea*	*Nocardia crassostrea*	*Crassostrea gigas*, *Ostrea edulis*	*rpoB*, *hsp65*, *gyrB*	3	400
*Marteilia refringens*	*Marteilia refringens*	*Ostrea edulis*, *Mytilus edulis*, *M*. *galloprovincialis*	ITS1O, ITS1M, probe	3	400
*Bonamia ostreae*	*Bonamia ostreae*	*Ostrea edulis*	5.8S‐ITS rDNA, *hsp90*, *act1*	3	400
OsHV‐1	Ostreid herpesvirus 1	*Crassostrea gigas*	C2/C6 (2), IA1‐IA2, orf4, Hyp. Protein, RING finger protein gene	7	400
			ORF100	1	198
			C9‐C10	1	197
			B3‐B4	1	207
*Enterococcus* spp.	*E*. *faecalis*, *E*. *faecium*, *E*. *avium*, *E*. *gallinarum*, *E*. *casseliflavus*, *E*. *durans*, *E*. *raffinosus*, *E*. *mundtii*	Human	*atpA*	8	400
*Roseovarius crassostrea*	*Roseovarius crassostrea*	*Crassostrea virginica*	*dnaJ*, *pyrH*	6	400
*Escherichia coli*	*Escherichia coli*	Human	*dnaJ*, *pyrH*, *atpA*, *gyrB*	4	400
*Aspergillus sydowii*	*Aspergillus sydowii*	*Gorgonia ventalina*, Human	TUB2, *trpC*, ITS, calmodulin gene	4	400
*Aurantimonas coralicida*	*Aurantimonas coralicida*	Corals	*atpD*, *gyrB*, *recA*, *rpoB*	4	400
*Serratia marcescens*	*Serratia marcescens*	*Acropora palmata*	*gyrB*, *recA*, *dnaJ*	3	400
*Pseudoalteromonas* sp.	*Pseudoalteromonas* sp.	*Rhopaloeides odorabile*	*gyrB*	1	400
			Total	884	29,292

**Figure 3:**
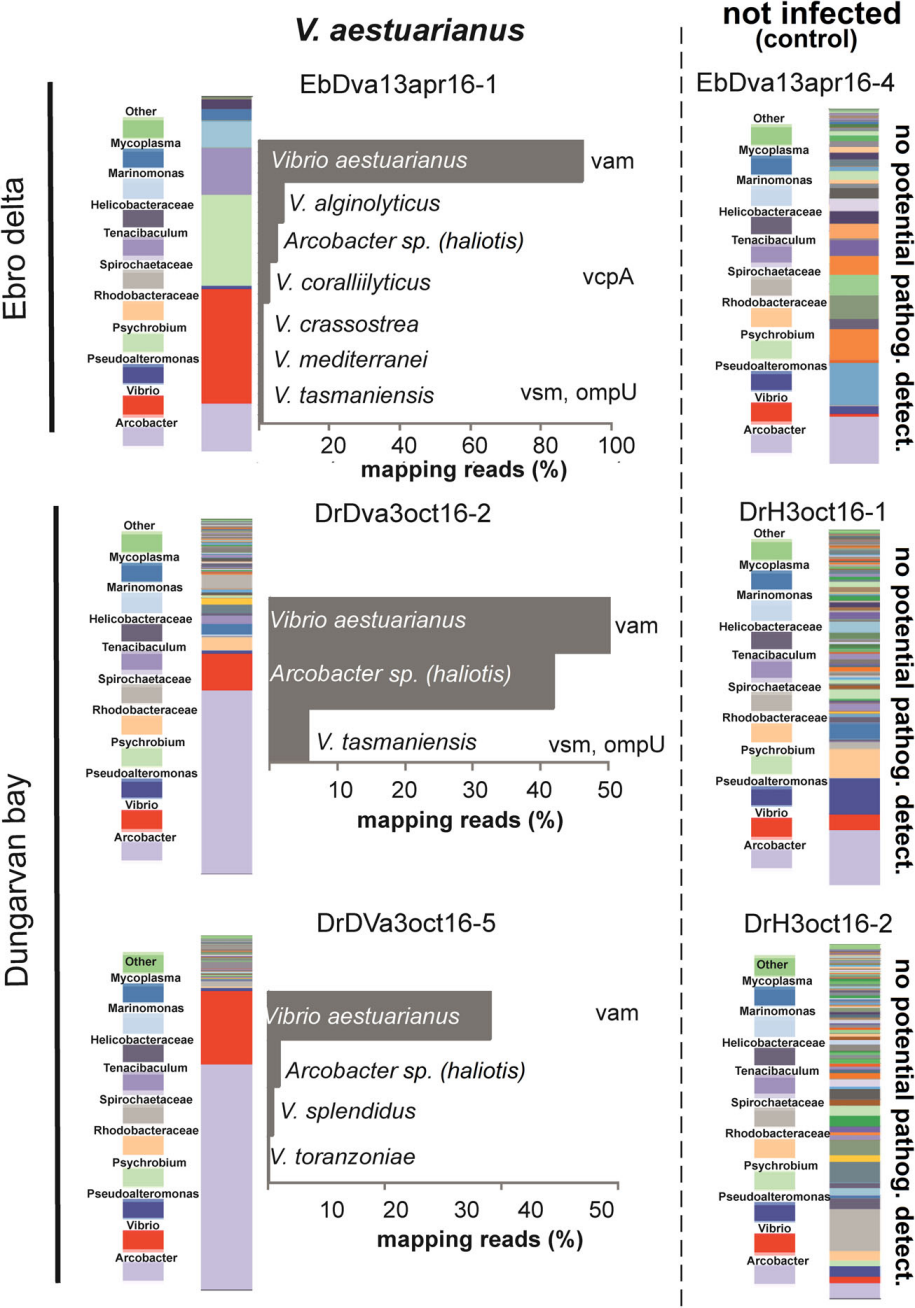
Results from target enrichment NGS analysis investigating *C*. *gigas* pathobiota in contrasting samples infected or not infected (control) by the bacteria *V*. *aestuarianus*. Relative abundance is calculated from the number of reads specifically mapping on target sequences and expressed as percentage (see main text for details on mapping parameters settings). Presence of virulence genes is also shown. [Color figure can be viewed at wileyonlinelibrary.com]

**Figure 4:**
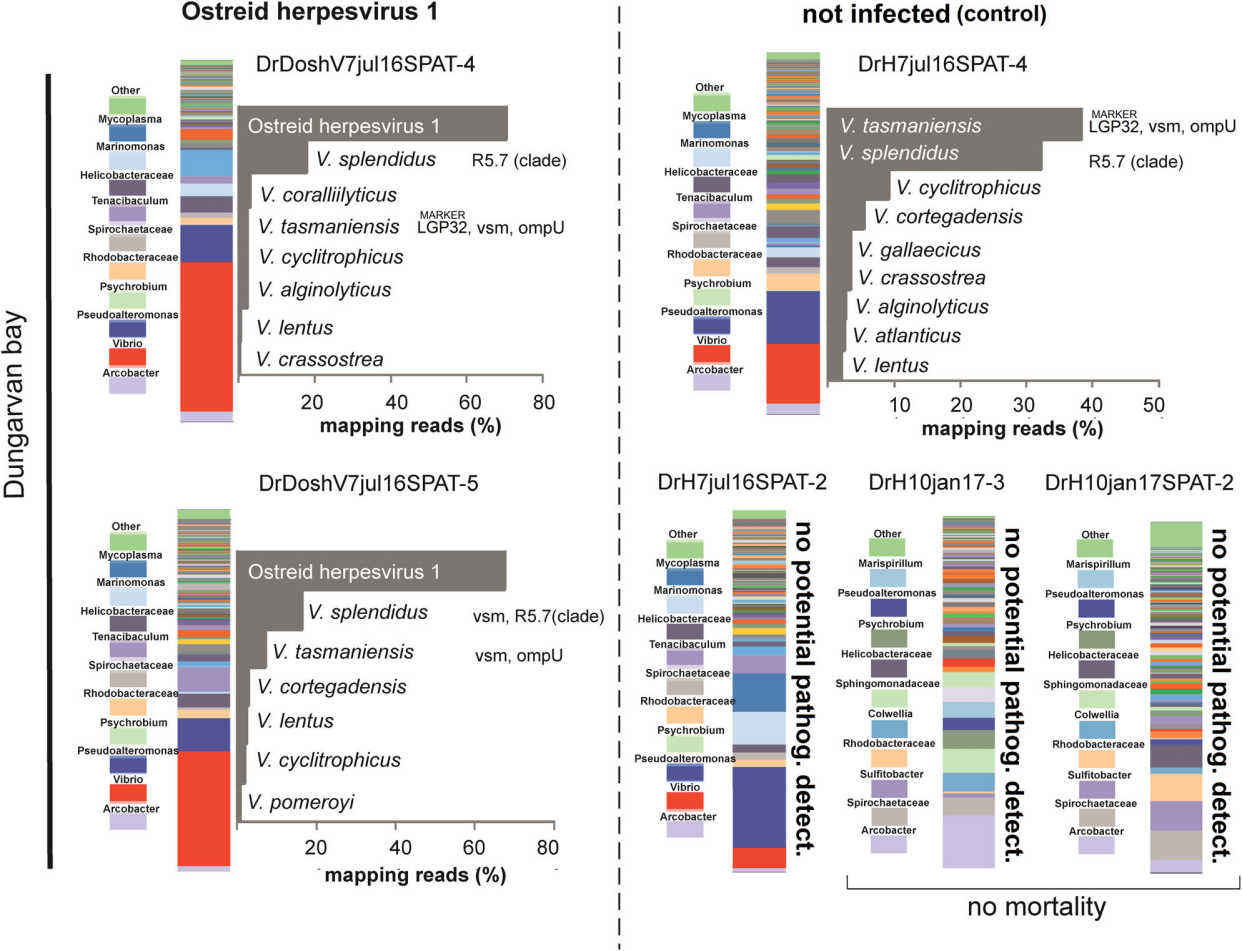
Results from target enrichment NGS analysis investigating *C*. *gigas* pathobiota in contrasting samples infected or not infected (control) by Ostreid herpesvirus 1 (OshV1). Samples DrH10jan17‐3 and DrH10jan17‐SPAT2 were collected during period of no mortality. Relative abundance is calculated from the number of reads specifically mapping on target sequences and expressed as percentage (see main text for details on mapping parameters settings). Presence of virulence genes is also shown. [Color figure can be viewed at wileyonlinelibrary.com]

Results of the target enrichment protocol allowed the detection and relative quantification of members of the bivalve pathogen community (pathobiota) associated to oyster tissues with a taxonomic resolution up to the species level. Generally, although the oyster pathobiota was composed of different species in all samples a dominance of primary pathogens such as *V*. *aestuarianus* and OsHV‐1 was observed in *C*. *gigas* samples collected during mortality episodes linked to these pathogens (Figs 3 and 4).

In particular, adult *C*. *gigas* samples collected during mortality episodes linked to *Vibrio aestuarianus* outbreaks in Ebro Delta and Dungarvan bay (e.g. samples EbDva13apr16‐1, DrDva3oct16‐2, DrDva3oct16‐5) showed a large proportion of reads specifically mapping on *V*. *aestuarianus* phylogenetic (e.g. *gyrB*, *recA*, *atpA*, *dnaJ* and *pyrH*) and virulence (e.g. *vam)* marker sequences accounting on average for more than 40% of total read sequences. Accordingly, consensus sequences obtained from read mapping were unequivocally assigned to the species *V*. *aestuarianus* by BLAST sequence analysis (Fig. 3). In addition, a significant fraction of reads common to all *V*. *aestuarianus* infected samples were specifically mapping on *Arcobacter haliotis* marker sequences (*gyrB*). Albeit at lower relative abundance other species identified in these samples included *V*. *alginolyticus*, *V*. *coralliilyticus*, *V*. *crassostreae*, *V*. *mediterranei*, *V*. *toranzoniae*, *V*. *splendidus* and *V*. *tasmaniensis* (Fig. 3). Virulence genes such as metalloproteases (*V*. *splendidus vsm*, *V*. *coralliilyticus vcpA*) and *ompU* of *V*. *tasmaniensis* were also detected (Fig. 3).

Similarly, *C*. *gigas* samples collected during mortality episodes of spat oysters linked to an OsHV‐1 outbreak in Dungarvan bay (e.g. samples DrDoshV7jul16SPAT‐4, DrDoshV7jul16SPAT‐5) showed a large number of reads (on average more than 60% of the total read sequences) specifically mapping on OsHV‐1 marker sequences (e.g. C2/C6 (2), IA1‐IA2, orf4, Hyp. Protein, RING finger protein gene, ORF100, C9‐C10, B3‐B4). An additional fraction of reads found in these samples pointed to the presence of bacterial species belonging to the Splendidus clade including *V*. *splendidus*, *V*. *tasmaniensis*, *V*. *cyclitrophicus* and *V*. *lentus*. Other bacterial species found in OsHV‐1 infected oyster included *V*. *coralliilyticus*, *V*. *alginolyitucs*, *V*. *crassostreae*, *V*. *cortegadensis* and *V*. *pomeroyi* (Fig. 4). Virulence genes (e.g. *V*. *tasmaniensis vsm* and *ompU*) and the genetic element R5.7 were also found (Fig. 4).

Generally, no reads were observed to map on reference sequences above defined thresholds in control samples, suggesting that target bacterial concentrations were either absent or below the limit of detection in these samples (Figs 3 and 4). An exception to this was observed for sample DrH7jul16SPAT‐4 collected in Dungarvan Bay during an OsHV‐1 outbreak in July 2016 where a diversified pathobiota community dominated by members of the Splendidus clade such as *V*. *splendidus* and *V*. *tasmaniensis* was found (Fig. 4). Virulence genes (e.g. *vsm* and *ompU*) and the genetic element R5.7 were detected in this sample as well as a number of other *Vibrio* species including *V*. *cyclitrophicus*, *V*. *cortegadensis*, *V*. *gallaecicus*, *V*. *crassostreae*, *V*. *alginolyticus*, *V*. *atlanticus* and *V*. *lentus* (Fig. 4). In contrast, no target sequences were detected in samples DrH10jan17SPAT‐2 and DrH10jan17‐3 collected in Dungarvan bay in winter in the absence of significant mortality.

All species included in the mock community sample were correctly identified by the target enrichment protocol developed in this study (Supplementary Material and Methods, Fig. S3), whilst no sequences were obtained from analysis of the negative control ruling out laboratory contamination.

## Discussion

### *Microbiota composition of healthy* C. gigas *oysters in European farming sites*

The composition of *C*. *gigas* microbiota in this study was dominated by the classes of *Gamma* and *Alphaproteobacteria* and was highly variable in relation to health status, geographic location, season and oyster age (Fig. 2). Accordingly, many transient and opportunistic microbial taxa appeared to dominate oyster microbiota while core microbial communities were restricted to only a few, most probably resident bacteria, such as *Vibrio* and the uncultured‐053 group. This may be linked to the filter feeding behaviour of oysters, which expose the animals to colonization by complex and highly variable microbial communities found in the seawater environment. *Mycoplasmataceae*, *Arcobacter*, *Synechococcus* and *Spirochaetaceae* dominated the microbiota in healthy (non‐infected) oysters suggesting these taxa might play a beneficial role in oyster fitness and health status (King *et al*., 2019). Interestingly, *Arcobacter* and *Mycoplasmataceae* were found to represent an abundant fraction of the microbiota in the Eastern oyster (*Crassostrea virginica*) and Chilean oyster (*Tiostrea chilensis*) respectively, although their role within the host is still largely unknown (Romero *et al*., 2002; King *et al*., 2012). Similarly, *Synechococcus* was observed in the cytosol of the digestive gland, connective tissue, mantle, and gonad of *C*. *gigas* and a host‐endobiont relationship between *C*. *gigas* and *Synechococcus* cells was recently suggested (Avila‐Poveda *et al*., 2014). In addition, since cyanoprokaryotes are particularly abundant in the digestive gland (Avila‐Poveda *et al*., 2014) and colonize the oyster microbiota especially in the summer period when seawater concentrations of these bacteria are higher (Fig. 1), it can be speculated that their accumulation in oyster tissues may directly derive from the natural environment. Host‐associated spirochetes have been found mainly in the digestive tract of eukaryotes including molluscs (Romero and Espejo, 2001; Duperron *et al*., 2007) but again little is known on their association and role with their *C*. *gigas* hosts.

### *Changes in the microbiota community structure associated to* C. gigas *infections during mass mortality episodes in Europe*

Comparative analysis of healthy versus infected *C*. *gigas* samples clearly showed that infected oysters displayed signs of community structure disruption and were characterized by a low diversity and proliferation of few bacterial taxa. This was particularly evident for *V*. *aestuarianus*‐infected oysters where dominance by bacteria belonging to the genus *Vibrio* and *Arcobacter* resulted in low microbial diversity compared with healthy oysters. In the case of OsHV‐1 infected oyster, loss of Alpha diversity was less clear and mostly linked to proliferation of *Vibrio* and a significant decline of some bacterial taxa such as cyanoprokaryotes (e.g. *Synechococcus*) (Fig. 1). Loss of microbiota diversity and proliferation of few OTUs (‘dysbiosis’) has previously been linked with impaired health in oysters (Green and Barnes, 2010; King *et al*., 2019), including *C*. *gigas* (Garnier *et al*., 2007; Lokmer and Wegner, 2015). It is thus apparent that specific microbial taxa and especially members of the *Vibrio* genus are likely to play a role in affecting oyster health status during disease outbreaks. Nevertheless, whether the condition of ‘dysbiosis’ is a prerequisite for oyster infection or it is a consequence of developing disease it is difficult to discern.

Recently an elegant study carried out by de Lorgeril *et al*. (2018) using experimental inoculations reproducing the natural route of infection in contrasting (susceptible vs. resistant) oyster families showed that the disease is caused by multiple infection with an initial and necessary step of infection of oyster haemocytes by the Ostreid herpesvirus OsHV‐1 μVar and subsequent bacteraemia by opportunistic bacteria. Accordingly, Lemire *et al*. (2015) showed that the onset of disease in oysters is associated with progressive replacement of diverse benign bacterial colonizers by members of a phylogenetically coherent virulent population. According to these results ‘dysbiosis’ might be seen as a new form of polymicrobial disease, in which a population/consortium of virulent strains but also non‐pathogenic strains contribute to oyster mortality (Lemire *et al*., 2015).

### *Potential pathogenic microbial communities (pathobiota) of* C. gigas *assessed through a new target enrichment next‐generation sequencing approach*

A larger number of microbial pathogens than previously thought might be involved in the establishment of oyster infection and development of diseases. Nevertheless, current knowledge in this field is restricted to culturable microorganisms (Lemire *et al*., 2015) most probably providing an incomplete view of the microbial communities potentially structuring the oyster pathobiota. Culture independent techniques such as 16S rRNA profiling or shotgun metagenomics widely employed in microbiome studies would also not be of great help to this purpose as they lack phylogenetic resolution (e.g. 16S rRNA gene‐based analysis) and/or might be hampered by large amounts of host DNA (Nhung *et al*., 2007; Forbes *et al*., 2017). For instance, *C*. *gigas* haemolymph is composed of approximately 10^6^ bacterial cells ml^−1^ (average bacterial genome size = 5 × 10^6^ bp) and typically in the order of 2 × 10^6^ haemocytes cells ml^−1^ (genome size = 8.2 × 10^8^ bp) resulting in a genomic content (calculated as the sum of bacterial plus host genomic DNA) of up to 1.6 × 10^15^ bp ml^−1^ of haemolymph (Hedgecock *et al*., 2005; Vezzulli *et al*., 2015). Considering that high‐performance sequencing technologies (e.g. NovaSeq 6000 System) might provide output up to 3 × 10^12^ bp per single run (2 × 10^10^ reads with 150 bp average read length), it can be theoretically calculated that shotgun metagenomic approaches might provide very low coverage (<0.002×) of metagenomic DNA derived from oyster tissues. In other words, in order to detect target genetic traits such as the bacteria phylogenetic or virulence markers investigated in this study with shotgun metagenomics, we might require them to be present at a concentration of at least 10^3^ (i.e. at least 2× coverage of the target region) in the analysed samples. Such concentrations probably need to be higher considering analysis bias such as non‐optimal sequencing performance, and the fact that whole tissue homogenate might show a greater genomic content than the one estimated above for theoretical calculations in oyster haemolymph.

To overcome these constrains, a new target enrichment next‐generation sequencing approach was developed and successfully applied to explore the Pacific Oyster pathobiota during mortality episodes in Europe. The protocol is based on the use of biotinylated RNA baits (on average >100‐mer) for selective capturing of 884 phylogenetic and virulence markers targeting the *Vibrio* community and other potential pathogenic microorganisms in oyster tissues via hybridization (Table 2). This approach is estimated to increase target DNA coverage by about three orders of magnitude compared with current approaches such as shotgun metagenomics (Vezzulli *et al*., 2017).

Generally, although the pathobiota community was composed of different microbial species in infected *C*. *gigas* samples a dominance of primary pathogens such as *V*. *aestuarianus* and OsHV‐1 was apparent (Figs 3 and 4). *Crassostrea gigas* samples infected by *V*. *aestuarianus* collected during infection outbreaks ascribed to this pathogen, showed the presence, albeit at lower relative abundance, of other potential pathogenic vibrios species including *V*. *coralliilyticus*, *V*. *splendidus*, *V*. *crassostreae*, *V*. *tasmaniensis* and their associated virulence genes (*vsm*, *vcpA* and *ompU*) (Fig. 3). These are likely opportunistic bacteria known to take advantage of a compromised host health status linked to infection and/or occurrence of stressful events (e.g. temperature stress) (Vezzulli *et al*., 2010). Accordingly, seawater temperature greater than 15°C is known to promote *Vibrio* replication in coastal marine waters, a condition commonly found during periods of mortality (e.g. summer) (Vezzulli et al., 2003; Stauder *et al*., 2010).

Interestingly, an *Arcobacter* sp. strain phylogenetically related to the species *Arcobacter haliotis* was consistently found in *V*. *aestuarianus*‐infected *C*. *gigas* samples collected both in the Ebro delta and Dungarvan bay. *Arcobacter* spp. strains have been previously found in association with the oyster haemolymph and proposed to represent specific symbionts of *C*. *gigas* (Lokmer and Wegner, 2015). Nevertheless, bacteria belonging to this genus were also observed in high abundance in moribund oysters, suggesting they might also turn into opportunistic pathogens at high density (Lokmer and Wegner, 2015). In this study, *Arcobacter* spp. represent an important fraction of the oyster microbiota whose relative abundance significantly increased in concomitance with V. aestuarianus infection. The species *Arcobacter haliotis* was isolated from the gut of an abalone of the species *Haliotis gigantea* collected in Japan (Tanaka *et al*., 2017), however, taxonomy of the *Arcobacter* sp. strain found in this study and its potential role as an opportunistic pathogen for *C*. *gigas* deserve further investigations (Pérez‐Cataluña *et al*., 2018).

The pathobiota community in OsHV‐1 infected oysters was also composed of a variety of potentially pathogenic *Vibrio* species most of which belong to the Splendidus super‐clade, e.g., *V*. *splendidus*, *V*. *tasmaniensis* (including the LGP32 pathogenic strain), *V*. *cyclitrophicus*, *V*. *lentus*, *V*. *pomeroyi* (Fig. 4) and other *Vibrio* species including *V. coralliilyticus*, *V*. *alginolyticus* and *V*. *cortegadensis*. Such observations add to previous findings from culture‐based studies investigating *Vibrio* microbiota in naturally infected specific‐pathogen‐free oysters by direct culturing on selective media (Lemire *et al*., 2015). Bacterial members of the Splendidus clade colonizing oyster tissues were found to belong to a phylogenetically coherent virulent population that may share virulence factors needed for oyster infection (Lemire *et al*., 2015). Accordingly, population‐specific genomic regions such as the R5.7 genetic element that was shown to play a role in oyster infection was detected in the microbiota of OsHV‐1‐infected oysters in this study. Other major virulence genes such as those encoding for *V*. *splendidus* and *V*. *tasmaniensis* metalloproteases (*vsm*) and outer membrane proteins (*ompU*) were also present.

In contrast to the above findings, analysis of healthy (non‐infected) oysters by target enrichment protocols failed to detect potential pathogenic species and their associated virulence genes in the majority of the samples. This result contrasts with the fact that members of the potential pathogenic microbial community such as genus *Vibrio* and *Arcobacter* were commonly found in control samples by 16S rRNA profiling analysis. Such a discrepancy could be linked to the low abundance of these bacterial groups in control samples compared to infected samples such that their presence might simply go undetected by target enrichment analysis. An exception is represented by sample DrH7jul16SPAT‐4 collected in Dungarvan Bay during an OsHV‐1 outbreak in July 2016. Even though this sample was not infected by OsHV‐1, it showed a diversified pathobiota community similar to the one observed in OsHV‐1‐infected oysters (Fig. 4). Members of the Splendidus clade including the oyster pathogen *Vibrio tasmaniensis* LGP32 strain dominated such communities; virulence genes (e.g. *vsm* and *ompU*) and the genetic element R5.7 were also present. It should be mentioned that not all *V*. *tasmaniensis* and *V*. *splendidus* are pathogenic. Considering that this oyster did, however, belong to the same cohort of oysters that were infected, it can be speculated that such results might reflect some early stage of infection before OsHV‐1 DNA reached a threshold above which it can be detected.

## Conclusions

Diseases of the Pacific oyster (*C. gigas*) associated with microbial infections have been rising over the past decades, representing a significant threat for the aquaculture production of the Pacific oysters in Europe. Stress‐induced changes in the composition of the oyster microbiota linked to compromised immune functions are thought to be responsible for disease development by replacement of benign microbial colonizers by consortia of different pathogens (pathobiota). In this study, the composition of the oyster pathobiota associated to two diseases (e.g. OsHV‐1 and *V*. *aestuarianus* infections) affecting the Pacific oysters at different life stages was for the first time assessed by employing 16S rRNA gene profiling and a new target enrichment next‐generation sequencing approach able to detect and relatively quantify potential pathogenic species including not culturable bacteria in oyster tissues. It was shown that primary infectious agents such as the bacteria *V*. *aestuarianus* and *Ostreid herpesvirus‐1* (OsHV‐1) dominated the pathobiota community of infected animals during mortality outbreak ascribed to these pathogens together with a previously undetected community of potential pathogenic bacterial species mostly belonging to the genus *Vibrio* and *Arcobacter*.

Our results provide, for the first time, full insight into the species/strain composition of the potential pathogenic microbial community associated to oyster tissues during mortality episodes. It is suggested that the biology and ecology of detected microbial species and their consortia should be targeted by future studies aimed to shed light on mechanisms underlying polymicrobial infections in *C*. *gigas*. More generally, the developed protocol and approach may be of great interest in monitoring oyster disease dynamics as well as in studies investigating infection diseases in other marine organisms and the biology and ecology of marine microbial pathogenic communities.

## Experimental procedures

### Crassostrea gigas *samples collection during mortality episodes in Europe*

In the frame of the EU funded H2020 project VIVALDI (Preventing and mitigating farmed bivalve diseases) 525 *C*. *gigas* individuals (365 spat and 160 adult) were collected between March 2016 and October 2017 at different European sites (Ebro delta, lat 40°29′37.7″N – long 0°48′24.82″E, Spain; Dungarvan Bay, lat 52°4′1.35″N – long 7°33′51.74″W, Ireland; Bay of Brest, lat 48°20′24.94″N – long 4°29′15.39″W, France) experiencing mass mortality episodes (Table 1, Supplementary Material and Methods, Fig. S1). Immediately after collection, individual oyster samples were transported to the laboratory and prepared for downstream molecular analysis according to EU Council Directive 175/2010. Briefly, bivalve tissues were extracted from single specimen and placed on a 2 ml tube containing beads, and frozen at −20°C until processed. Samples were placed on 180 μl of ATL buffer and 20 μl of proteinase K to be homogenized, followed by DNA extraction using Blood and Tissue Kit (QIAGEN srl, Milan, Italy), following the instructions for Tissue Protocol. The amount of extracted DNA was quantified using the Quantifluor double‐stranded DNA quantification kit (Promega Italia, Milan, Italy).

### *OsHV‐1 and* Vibrio aestuarianus *detection in* C. gigas *samples by qPCR*

Individual oyster samples were preliminary screened for the presence of OsHV‐1 and *Vibrio aestuarianus* by real‐time PCR as previously described (Webb *et al*., 2007; IFREMER, 2013). Briefly, for detection of OsHV‐1 real‐time PCR was performed using primers HVDP‐F ATTGATGATGTGGATAATCTGTG and HVDP‐R GGTAAATACCATTGGTCTTGTTCC according to Webb *et al*. (2007). Amplification was performed using a ABI 7300 Thermocycler (Applied Biosystems, CA) in a total volume of 20 μl. The PCR mix included 1 μl of extracted DNA, 10 μl 2× SYBR GREEN dye, 0.50 μl of each diluted primers (final concentration 0.5 μM) and 8 μl of molecular grade water. Plasmid DNA containing cloned viral DNA was used as a positive control. Quantification of OsHV‐1 DNA copies was carried out using a standard curve based on 10‐fold dilutions of OsHV‐1 genomic DNA.

For detection of *V*. *aestuarianus*, a Taqman real‐time PCR protocol with the LightCyler (Roche Diagnostics, Mannheim, Germany) was used. *Vibrio aestuarianus* specific primers and probe (DNAj F GTATGAAATTTTAACTGACCCACAA; DNAj R CAATTTCTTTCGAACAACCAC; DNAj probe FAM– TGGTAGCGCAGACTTCGGCGAC – BHQ2) (IFREMER, 2013) were used in the assays. Each reaction mixture contained 1× LightCycler Taqman master (Roche Diagnostics, Mannheim, Germany) and 1 μM of each primer and 0.1 μM of each probe in a final volume of 20 μl. Five microliters of DNA template (DNA concentration for all samples varied from 10 to 100 ng μl^−1^) was added to the reaction mixture. Accurately quantified copy number genomic DNA of *V*. *aestuarianus* 01/32 strains was used as a standard. Positive and negative controls (PCR grade water, Sigma Aldrich S.R.L., Milan) were included in all qPCR assays. Presence/absence of the targeted pathogens in the analysed samples is presented in this study.

### *Analysis of bivalve ‘microbiota’ by 16S rRNA gene‐based profiling of the bacterial community*

16S rDNA PCR amplicon libraries were generated from genomic DNA extracted from individual bivalve samples using primers amplifying positions 515–802 of the *Escherichia coli* numbering of the 16S rRNA gene that include the V4 hypervariable region. All primers were custom designed to include 16S rRNA complementary regions plus the complementary sequences to the Ion Torrent specific adapters. Two PCR assays were performed. A first target enrichment PCR assay with the 16S conserved primers (Supplementary Material and Methods, Table S1). A second PCR assay, with customized primers including adapters' complementary regions (Supplementary Material and Methods, Table S1). The obtained libraries were sequenced using an Ion Torrent (PGM) Platform (Thermo Fisher Scientific, MA).

Bioinformatic analysis of NGS data was performed using the Microbial Genomics module (version 1.3) workflow of the CLC Genomics workbench (version 9.5.1) and other comparable software. After quality trimming based on quality scores and length trimming, reads were clustered at 97% level of similarity into OTUs. Chimera detection and removal was performed. Ribosomal RNA gene reads were classified against the non‐redundant version of the SILVA SSU reference taxonomy (release 123; http://www.arb-silva.de). Only reads occurring at least five times in the trimmed data set were assigned to bacterial taxa and included in the results.

Alpha diversity analysis was then conducted on total OTUs by constructing rarefaction curves calculated by sub‐sampling OTUs abundances in the different samples at different depths. Beta diversity analysis was also performed by calculating Bray–Curtis distances between each pair of samples and applying Principal Coordinate Analysis on the distance matrices.

The core microbiota, defined as the microbial taxa belonging to OTUs present in all the samples, was analysed using the Corbata software (CORe microbiome Analysis Tools) (Li *et al*., 2013). A two‐parameter model (Ubiquity‐Abundance) was applied to quantitatively identify the core taxonomic members of each sample group microbiota considering the different conditions. Sequence reads data were archived at NCBI sequence read archive (SRA) with Accession Number PRJNA542081.

### *Target enrichment next‐generation sequencing protocol for the analysis of the bivalve Pathobiota*

A target enrichment next‐generation sequencing protocol for the analysis of bivalve ‘pathobiota’ was applied following the approach previously developed for the target sequencing of *Vibrio cholerae* DNA in complex environmental samples (Vezzulli *et al*., 2017). To this aim, 884 *Vibrio* phylogenetic and virulence markers, as well as other bivalve and marine invertebrates microbial pathogen markers (Rosenberg *et al*., 2007; Travers *et al*., 2015) with average length of 400 nt, were identified and used to produce 100‐mer biotinylated RNA baits for selective capturing of target DNA markers via hybridization (Table 2). Five hundred nanogram of total RNA baits was produced using the MYcroarray target enrichment proprietary technology (MYcroarray, Ann Arbor, MI) and used for a capture (it is estimated that a single capture is capable of enriching single‐copy nuclear loci, i.e., >99.5% of the capture target region) (Vezzulli *et al*., 2017).

Genomic DNA extracted from individual bivalve samples was sized on an Agilent Bioanalyzer and enzymatically fragmented using the KAPA Frag Kit (Roche Diagnostics, Mannheim, Germany) protocol to an average size of about 600 bp. The fragmented DNA was used for the production of an indexed library for next‐generation sequencing on the Illumina platform (Illumina) using the KAPA HyperPlus Kit for Illumina (Roche Diagnostics). About 200 ng of the produced library was used for target DNA capturing using the MYbaits protocol (MYcroarray, Ann Arbor, MI) following the manufacturers instructions. Briefly, the genomic DNA library was heat‐denatured and hybridized to the RNA baits in stringent conditions. Hybridization was carried out at 65°C for 36H (capturing of target DNA with 5%–10% sequence divergence is expected at this conditions enabling full covering of marker allelic variants including unknown variants). After hybridization, the biotinylated baits hybridized to captured material were pulled out of the solution with streptavidin‐coated magnetic beads and the captured genomic DNA was released by chemical degradation of the RNA baits. Enriched libraries were amplified prior to sequencing.

All samples libraries were then pooled and sequenced on a MiSeq Illumina™ platform (V3 flow cell, 600 cycles, 25 M reads 250 bp pair ends). After quality trimming, sequence reads were mapped against reference sequences of phylogenetic and virulence markers used to produce the baits using the CLC mapping tool set with length fraction of 0.5 and similarity fraction of 0.95. Consensus sequences were produced from outputs with a minimum of 10 reads mapping on the reference sequences and blasted against the nucleotide collection (nr) database of NCBI for classification.

In addition to bivalve samples, DNA extracted from a mock community sample (positive control) composed of equal amount of genomic DNA from *Vibrio cholerae* O139 5424, *Vibrio tasmaniensis* LGP32, *Vibrio alginolyticus*, *Vibrio cholerae* non O1/O139, *Vibrio mimicus* CP192, *Vibrio cholerae* O1 CD81 classical, *Vibrio aestuarianus* 01/032,V*ibrio cholerae* N16961^T^ El Tor, *Vibrio coralliilyticus* ATCC BAA 450, *Vibrio tapetis* CECT 4600^T^, *Vibrio vulnificus* ATCC 275262, *Vibrio parahaemolyticus* 54496, *Escherichia coli* ATCC 2922, *Serratia marcescens*, *Enterococcus faecalis* ATCC 29212 and a nuclease‐free water sample (negative control) was also analysed and sequenced following the same protocol.

To avoid laboratory contamination of treated samples all the analyses including DNA extraction, DNA amplification and NGS library preparations were carried out in a separate laboratory (non‐aquatic/non‐microbiological laboratory) using a dedicated set of pipettes, reagents and consumables (Vezzulli *et al*., 2017). Sequence reads data were archived at NCBI SRA with accession: PRJNA542081.

## Conflict of Interest

The authors declare that they have no conflict of interests related to this work.

## Supporting information


**Figure S1.** Geographic areas and shellfish farms investigated in this study.

**Figure S2.** Rarefaction curves computed for total OTUs abundance (Alpha diversity analysis) (Va = *Vibrio aestuarianus* infected oysters; OshV1 = Ostreid herpesvirus 1 infected oysters).

**Figure S3.** Results from target enrichment NGS analysis targeting *C*. *gigas* pathobiota on a mock community sample (positive control) composed of equal amount of genomic DNA from *Vibrio cholerae* O139 5424, *Vibrio tasmaniensis* LGP32, *Vibrio alginolyticus*, *Vibrio cholerae* non O1/O139, *Vibrio mimicus* CP192, *Vibrio cholerae* O1 CD81 classical, *Vibrio aestuarianus* 01/032,V*ibrio cholerae* N16961^T^ El Tor, *Vibrio coralliilyticus* ATCC BAA 450, *Vibrio tapetis* CECT 4600^T^, *Vibrio vulnificus* ATCC 275262, *Vibrio parahaemolyticus* 54,496, *Escherichia coli* ATCC 2922, *Serratia marcescens*, *Enterococcus faecalis* ATCC 29212). Relative abundance is calculated from the number of reads specifically mapping on target sequences and expressed as percentage (see main text for details on mapping parameters settings).

**Table S1.** primers, adapter and barcode sequences used in this study.

**Table S2.** Core microbiota, shared genera in the different conditions at 0.1% of relative abundance and presence in, at least, 90% of the samples.

**Appendix S1:** Supplementary File.

## Data Availability

Sequence files and metadata for all samples and the mock community (positive control) used in this study have been deposited at NCBI SRA with accession: PRJNA542081. Additional data (Supplementary Material and Methods) including map of sampling areas, sequencing results from mock community analysis, primers used for 16S rRNA sequencing have all been included as additional supplementary files. 100‐merBaits sequences developed and used in the target enrichment protocol have also been included in supplementary material.
