# Supplementary figures and images for: Dynamics of the Pacific oyster pathobiota during mortality episodes in Europe assessed by 16S rRNA gene profiling and a new target enrichment next‐generation sequencing strategy
Source: Environ Microbiol. 2019 Jul 31;21(12):4548–62. doi: 10.1111/1462-2920.14750 (PMC7379488; doi:10.1111/1462-2920.14750)

**Figure S1.** Geographic areas and shellfish farms investigated in this study

**
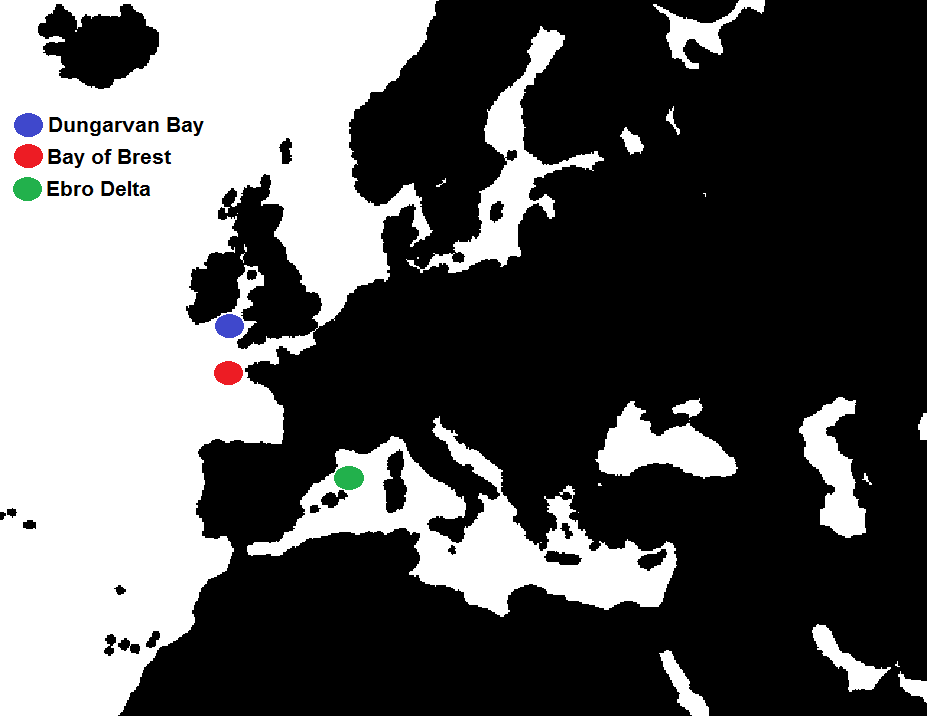
**

Supplement: Supplementary file 1 — Figure S1. Geographic areas and shellfish farms investigated in this study. [file EMI-21-4548-s001.docx]
