# Supplementary material for: Dynamics of the Pacific oyster pathobiota during mortality episodes in Europe assessed by 16S rRNA gene profiling and a new target enrichment next‐generation sequencing strategy
Source: Environ Microbiol. 2019 Jul 31;21(12):4548–62. doi: 10.1111/1462-2920.14750 (PMC7379488; doi:10.1111/1462-2920.14750)

**Figure S2.** Rarefaction curves computed for total OTUs abundance (Alpha diversity analysis) (Va=Vibrio aestuarianus infected oysters; OshV1= Ostreid herpesvirus 1 infected oysters)


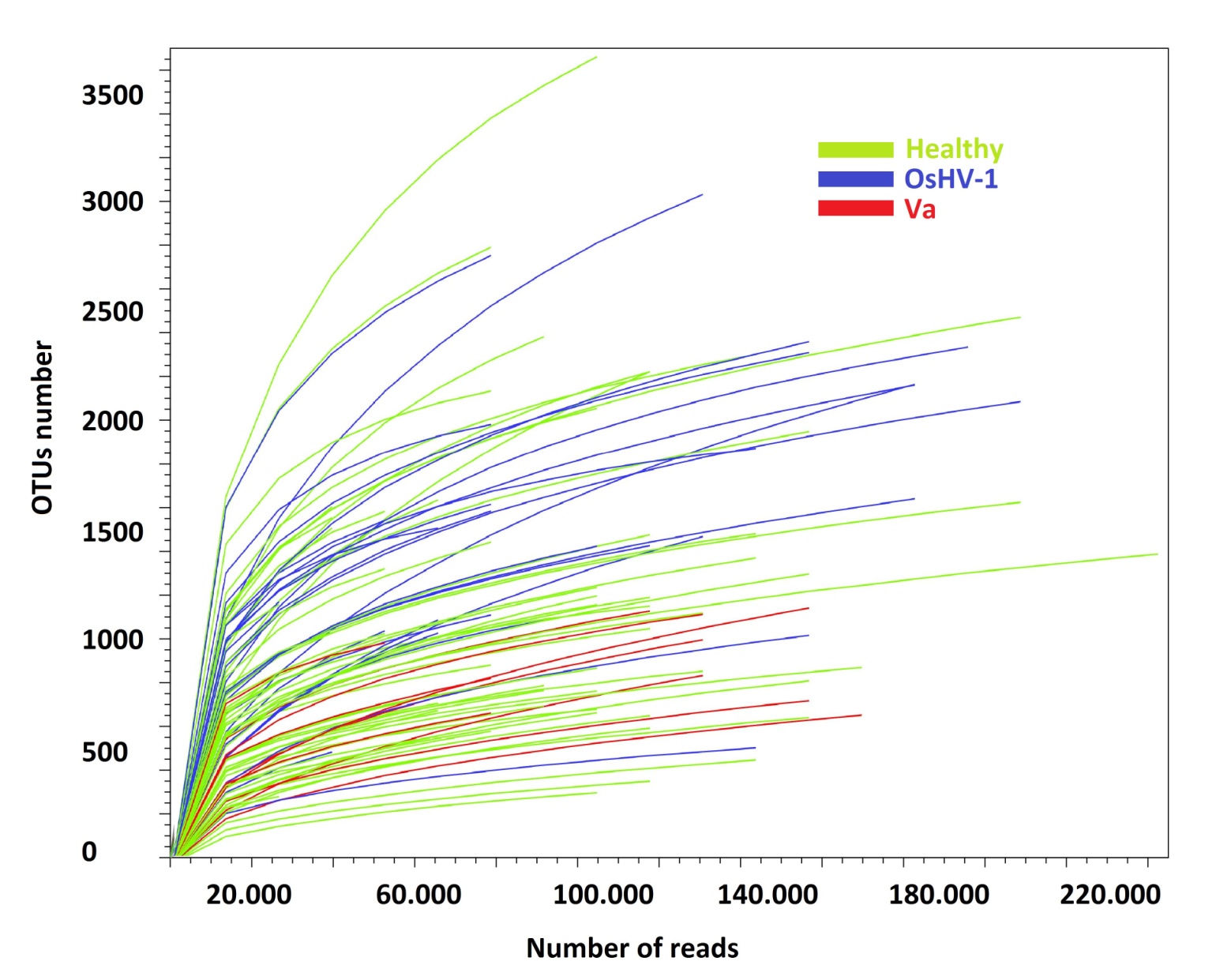

Supplement: Supplementary file 2 — Figure S2. Rarefaction curves computed for total OTUs abundance (Alpha diversity analysis) (Va = Vibrio aestuarianus infected oysters; OshV1 = Ostreid herpesvirus 1 infected oysters). [file EMI-21-4548-s002.docx]
