# Supplementary material for: Dynamics of the Pacific oyster pathobiota during mortality episodes in Europe assessed by 16S rRNA gene profiling and a new target enrichment next‐generation sequencing strategy
Source: Environ Microbiol. 2019 Jul 31;21(12):4548–62. doi: 10.1111/1462-2920.14750 (PMC7379488; doi:10.1111/1462-2920.14750)

**Figure S3.** Results from target enrichment NGS analysis targeting *C. gigas* pathobiota on a mock community sample (positive control) composed of equal amount of genomic DNA from *Vibrio cholerae* O139 5424, *Vibrio tasmaniensis* LGP32, *Vibrio alginolyticus, Vibrio cholerae* non O1/O139, *Vibrio mimicus* CP192, *Vibrio cholerae* O1 CD81 classical, *Vibrio aestuarianus* 01/032,V*ibrio cholerae* N16961^T^ El Tor, *Vibrio coralliilyticus* ATCC BAA 450, *Vibrio tapetis* CECT 4600^T^, *Vibrio vulnificus* ATCC 275262, *Vibrio parahaemolyticus* 54496, *Escherichia coli* ATCC 2922, *Serratia marcescens, Enterococcus faecalis* ATCC 29212). Relative abundance is calculated from the number of reads specifically mapping on target sequences and expressed as percentage (see main text for details on mapping parameters settings).


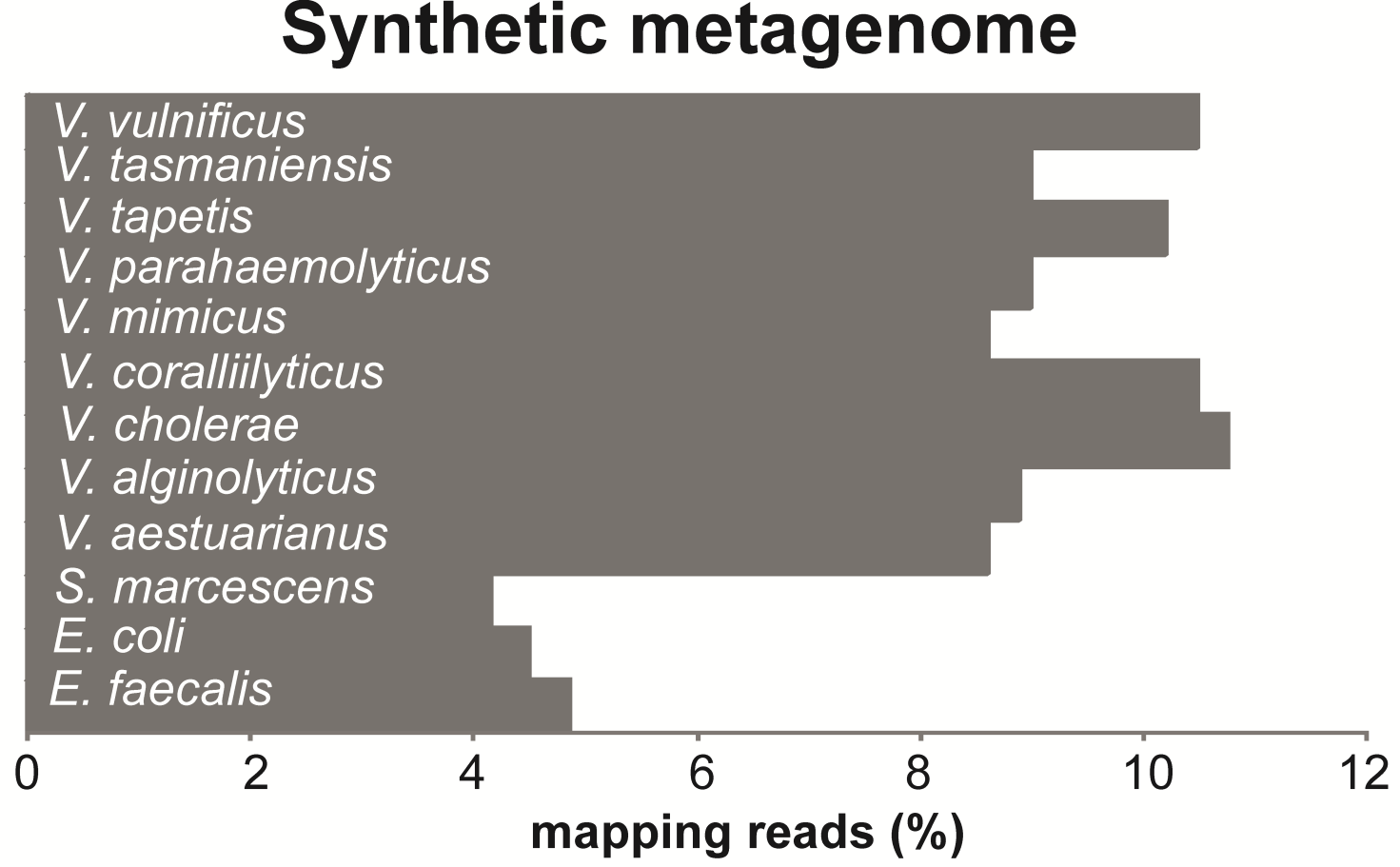

Supplement: Supplementary file 3 — Figure S3. Results from target enrichment NGS analysis targeting C. gigas pathobiota on a mock community sample (positive control) composed of equal amount of genomic DNA from Vibrio cholerae O139 5424, Vibrio tasmaniensis LGP32, Vibrio alginolyticus, Vibrio cholerae non O1/O139, Vibrio mimicus CP192, Vibrio cholerae O1 CD81 classical, Vibrio aestuarianus 01/032,Vibrio cholerae N16961T El Tor, Vibrio coralliilyticus ATCC BAA 450, Vibrio tapetis CECT 4600T, Vibrio vulnificus ATCC 275262, Vibrio parahaemolyticus 54,496, Escherichia coli ATCC 2922, Serratia marcescens, Enterococcus faecalis ATCC 29212). Relative abundance is calculated from the number of reads specifically mapping on target sequences and expressed as percentage (see main text for details on mapping parameters settings). [file EMI-21-4548-s003.docx]
