# Supplementary material for: Dynamics of the Pacific oyster pathobiota during mortality episodes in Europe assessed by 16S rRNA gene profiling and a new target enrichment next‐generation sequencing strategy
Source: Environ Microbiol. 2019 Jul 31;21(12):4548–62. doi: 10.1111/1462-2920.14750 (PMC7379488; doi:10.1111/1462-2920.14750)
Supplement: Supplementary file 4 — Table S1. primers, adapter and barcode sequences used in this study. [file EMI-21-4548-s004.docx]

**Table S1**: primers, adapter and barcode sequences used in this study

**1 step amplification (V4 primers)**

V4 FORWARD:

U515F + Unitail1 CAGGACCAGGGTACGGTGGTGCCAGCMGCCGCGGTA A

V4 MIX OF REVERSE:

802 R + unitail 2 CGCAGAGAGGCTCCGTGTACNVGGGTATCTAATCC

806r_UNI2 CGCAGAGAGGCTCCGTGGACTACHVGGGTWTCTAAT

**2 step amplification (adapter and barcodes)**

| NAME | trP1 | UNITAIL_2 | |  |  |
| --- | --- | --- | --- | --- | --- |
| ION_UNI_trP1 | CCTCTCTATGGGCAGTCGGTGAT | CGCAGAGAGGCTCCGTG | |  |  |
|  |  |  |  |  |  |
| NAME | A | key | MID(BARCODE) |  | UNITAIL_1 |
| ION_UNI1_A_1 | CCATCTCATCCCTGCGTGTCTCCGAC | TCAG | CTAAGGTAAC | GAT | CAGGACCAGGGTACGGTG |
| ION_UNI1_A_2 | CCATCTCATCCCTGCGTGTCTCCGAC | TCAG | TAAGGAGAAC | GAT | CAGGACCAGGGTACGGTG |
| ION_UNI1_A_3 | CCATCTCATCCCTGCGTGTCTCCGAC | TCAG | AAGAGGATTC | GAT | CAGGACCAGGGTACGGTG |
| ION_UNI1_A_4 | CCATCTCATCCCTGCGTGTCTCCGAC | TCAG | TACCAAGATC | GAT | CAGGACCAGGGTACGGTG |
| ION_UNI1_A_5 | CCATCTCATCCCTGCGTGTCTCCGAC | TCAG | CAGAAGGAAC | GAT | CAGGACCAGGGTACGGTG |
| ION_UNI1_A_6 | CCATCTCATCCCTGCGTGTCTCCGAC | TCAG | CTGCAAGTTC | GAT | CAGGACCAGGGTACGGTG |
| ION_UNI1_A_7 | CCATCTCATCCCTGCGTGTCTCCGAC | TCAG | TTCGTGATTC | GAT | CAGGACCAGGGTACGGTG |
| ION_UNI1_A_8 | CCATCTCATCCCTGCGTGTCTCCGAC | TCAG | TTCCGATAAC | GAT | CAGGACCAGGGTACGGTG |
| ION_UNI1_A_9 | CCATCTCATCCCTGCGTGTCTCCGAC | TCAG | TGAGCGGAAC | GAT | CAGGACCAGGGTACGGTG |
| ION_UNI1_A_10 | CCATCTCATCCCTGCGTGTCTCCGAC | TCAG | CTGACCGAAC | GAT | CAGGACCAGGGTACGGTG |
| ION_UNI1_A_11 | CCATCTCATCCCTGCGTGTCTCCGAC | TCAG | TCCTCGAATC | GAT | CAGGACCAGGGTACGGTG |
| ION_UNI1_A_12 | CCATCTCATCCCTGCGTGTCTCCGAC | TCAG | TAGGTGGTTC | GAT | CAGGACCAGGGTACGGTG |
| ION_UNI1_A_13 | CCATCTCATCCCTGCGTGTCTCCGAC | TCAG | TCTAACGGAC | GAT | CAGGACCAGGGTACGGTG |
| ION_UNI1_A_14 | CCATCTCATCCCTGCGTGTCTCCGAC | TCAG | TTGGAGTGTC | GAT | CAGGACCAGGGTACGGTG |
| ION_UNI1_A_15 | CCATCTCATCCCTGCGTGTCTCCGAC | TCAG | TCTAGAGGTC | GAT | CAGGACCAGGGTACGGTG |
| ION_UNI1_A_16 | CCATCTCATCCCTGCGTGTCTCCGAC | TCAG | TCTGGATGAC | GAT | CAGGACCAGGGTACGGTG |
| ION_UNI1_A_17 | CCATCTCATCCCTGCGTGTCTCCGAC | TCAG | TCTATTCGTC | GAT | CAGGACCAGGGTACGGTG |
| ION_UNI1_A_18 | CCATCTCATCCCTGCGTGTCTCCGAC | TCAG | AGGCAATTGC | GAT | CAGGACCAGGGTACGGTG |
| ION_UNI1_A_19 | CCATCTCATCCCTGCGTGTCTCCGAC | TCAG | TTAGTCGGAC | GAT | CAGGACCAGGGTACGGTG |
| ION_UNI1_A_20 | CCATCTCATCCCTGCGTGTCTCCGAC | TCAG | CAGATCCATC | GAT | CAGGACCAGGGTACGGTG |
| ION_UNI1_A_21 | CCATCTCATCCCTGCGTGTCTCCGAC | TCAG | TCGCAATTAC | GAT | CAGGACCAGGGTACGGTG |
| ION_UNI1_A_22 | CCATCTCATCCCTGCGTGTCTCCGAC | TCAG | TTCGAGACGC | GAT | CAGGACCAGGGTACGGTG |
| ION_UNI1_A_23 | CCATCTCATCCCTGCGTGTCTCCGAC | TCAG | TGCCACGAAC | GAT | CAGGACCAGGGTACGGTG |
| ION_UNI1_A_24 | CCATCTCATCCCTGCGTGTCTCCGAC | TCAG | AACCTCATTC | GAT | CAGGACCAGGGTACGGTG |
| ION_UNI1_A_25 | CCATCTCATCCCTGCGTGTCTCCGAC | TCAG | CCTGAGATAC | GAT | CAGGACCAGGGTACGGTG |
| ION_UNI1_A_26 | CCATCTCATCCCTGCGTGTCTCCGAC | TCAG | TTACAACCTC | GAT | CAGGACCAGGGTACGGTG |
| ION_UNI1_A_27 | CCATCTCATCCCTGCGTGTCTCCGAC | TCAG | AACCATCCGC | GAT | CAGGACCAGGGTACGGTG |
| ION_UNI1_A_28 | CCATCTCATCCCTGCGTGTCTCCGAC | TCAG | ATCCGGAATC | GAT | CAGGACCAGGGTACGGTG |
| ION_UNI1_A_29 | CCATCTCATCCCTGCGTGTCTCCGAC | TCAG | TCGACCACTC | GAT | CAGGACCAGGGTACGGTG |
| ION_UNI1_A_30 | CCATCTCATCCCTGCGTGTCTCCGAC | TCAG | CGAGGTTATC | GAT | CAGGACCAGGGTACGGTG |
| ION_UNI1_A_31 | CCATCTCATCCCTGCGTGTCTCCGAC | TCAG | TCCAAGCTGC | GAT | CAGGACCAGGGTACGGTG |
| ION_UNI1_A_32 | CCATCTCATCCCTGCGTGTCTCCGAC | TCAG | TCTTACACAC | GAT | CAGGACCAGGGTACGGTG |
| ION_UNI1_A_33 | CCATCTCATCCCTGCGTGTCTCCGAC | TCAG | TTCTCATTGAAC | GAT | CAGGACCAGGGTACGGTG |
| ION_UNI1_A_34 | CCATCTCATCCCTGCGTGTCTCCGAC | TCAG | TCGCATCGTTC | GAT | CAGGACCAGGGTACGGTG |
| ION_UNI1_A_35 | CCATCTCATCCCTGCGTGTCTCCGAC | TCAG | TAAGCCATTGTC | GAT | CAGGACCAGGGTACGGTG |
| ION_UNI1_A_36 | CCATCTCATCCCTGCGTGTCTCCGAC | TCAG | AAGGAATCGTC | GAT | CAGGACCAGGGTACGGTG |
| ION_UNI1_A_37 | CCATCTCATCCCTGCGTGTCTCCGAC | TCAG | CTTGAGAATGTC | GAT | CAGGACCAGGGTACGGTG |
| ION_UNI1_A_38 | CCATCTCATCCCTGCGTGTCTCCGAC | TCAG | TGGAGGACGGAC | GAT | CAGGACCAGGGTACGGTG |
| ION_UNI1_A_39 | CCATCTCATCCCTGCGTGTCTCCGAC | TCAG | TAACAATCGGC | GAT | CAGGACCAGGGTACGGTG |
| ION_UNI1_A_40 | CCATCTCATCCCTGCGTGTCTCCGAC | TCAG | CTGACATAATC | GAT | CAGGACCAGGGTACGGTG |
| ION_UNI1_A_41 | CCATCTCATCCCTGCGTGTCTCCGAC | TCAG | TTCCACTTCGC | GAT | CAGGACCAGGGTACGGTG |
| ION_UNI1_A_42 | CCATCTCATCCCTGCGTGTCTCCGAC | TCAG | AGCACGAATC | GAT | CAGGACCAGGGTACGGTG |
| ION_UNI1_A_43 | CCATCTCATCCCTGCGTGTCTCCGAC | TCAG | CTTGACACCGC | GAT | CAGGACCAGGGTACGGTG |
| ION_UNI1_A_44 | CCATCTCATCCCTGCGTGTCTCCGAC | TCAG | TTGGAGGCCAGC | GAT | CAGGACCAGGGTACGGTG |
| ION_UNI1_A_45 | CCATCTCATCCCTGCGTGTCTCCGAC | TCAG | TGGAGCTTCCTC | GAT | CAGGACCAGGGTACGGTG |
| ION_UNI1_A_46 | CCATCTCATCCCTGCGTGTCTCCGAC | TCAG | TCAGTCCGAAC | GAT | CAGGACCAGGGTACGGTG |
| ION_UNI1_A_47 | CCATCTCATCCCTGCGTGTCTCCGAC | TCAG | TAAGGCAACCAC | GAT | CAGGACCAGGGTACGGTG |
| ION_UNI1_A_48 | CCATCTCATCCCTGCGTGTCTCCGAC | TCAG | TTCTAAGAGAC | GAT | CAGGACCAGGGTACGGTG |
| ION_UNI1_A_49 | CCATCTCATCCCTGCGTGTCTCCGAC | TCAG | TCCTAACATAAC | GAT | CAGGACCAGGGTACGGTG |
| ION_UNI1_A_50 | CCATCTCATCCCTGCGTGTCTCCGAC | TCAG | CGGACAATGGC | GAT | CAGGACCAGGGTACGGTG |
| ION_UNI1_A_51 | CCATCTCATCCCTGCGTGTCTCCGAC | TCAG | TTGAGCCTATTC | GAT | CAGGACCAGGGTACGGTG |
