# Supplementary material for: Dynamics of the Pacific oyster pathobiota during mortality episodes in Europe assessed by 16S rRNA gene profiling and a new target enrichment next‐generation sequencing strategy
Source: Environ Microbiol. 2019 Jul 31;21(12):4548–62. doi: 10.1111/1462-2920.14750 (PMC7379488; doi:10.1111/1462-2920.14750)
Supplement: Supplementary file 5 — Table S2. Core microbiota, shared genera in the different conditions at 0.1% of relative abundance and presence in, at least, 90% of the samples. [file EMI-21-4548-s005.docx]

**Table S2** Core microbiota, shared genera in the different conditions at 0.1% of relative abundance and presence in, at least, 90 % of the samples
